# Insect Pheromone Receptors – Key Elements in Sensing Intraspecific Chemical Signals

**DOI:** 10.3389/fncel.2018.00425

**Published:** 2018-11-20

**Authors:** Jörg Fleischer, Jürgen Krieger

**Affiliations:** Department of Animal Physiology, Institute of Biology/Zoology, Martin Luther University Halle-Wittenberg, Halle, Germany

**Keywords:** chemoreception, pheromone signaling, olfaction, odorant receptor, signal transduction

## Abstract

Pheromones are chemicals that serve intraspecific communication. In animals, the ability to detect and discriminate pheromones in a complex chemical environment substantially contributes to the survival of the species. Insects widely use pheromones to attract mating partners, to alarm conspecifics or to mark paths to rich food sources. The various functional roles of pheromones for insects are reflected by the chemical diversity of pheromonal compounds. The precise detection of the relevant intraspecific signals is accomplished by specialized chemosensory neurons housed in hair-like sensilla located on the surface of body appendages. Current data indicate that the extraordinary sensitivity and selectivity of the pheromone-responsive neurons (PRNs) is largely based on specific pheromone receptors (PRs) residing in their ciliary membrane. Besides these key elements, proper ligand-induced responses of PR-expressing neurons appear to generally require a putative co-receptor, the so-called “sensory neuron membrane protein 1” (SNMP1). Regarding the PR-mediated chemo-electrical signal transduction processes in insect PRNs, ionotropic as well as metabotropic mechanisms may be involved. In this review, we summarize and discuss current knowledge on the peripheral detection of pheromones in the olfactory system of insects with a focus on PRs and their specific role in the recognition and transduction of volatile intraspecific chemical signals.

## Introduction

Pheromone signals released from individuals to affect the behavior or physiology of conspecifics play a pivotal role for numerous animal species. In insects, pheromones trigger and control various critical processes such as mating, reproduction, aggregation and alarming as well as the division of labor in eusocial species (Wyatt, 2014). Pheromones are adequate stimuli of powerful chemosensory systems that enable insects to sensitively detect and discriminate the relevant compounds in a complex chemical world that surrounds them (Deisig et al., 2014; Renou, 2014).

Volatile pheromone molecules are generally detected through specialized sensory neurons of the olfactory system located on the antennae (Hansson and Stensmyr, 2011), whereas non-volatile pheromones are usually received by contact chemoreception mediated by neurons of the gustatory system that predominantly reside on the proboscis and legs (Ebbs and Amrein, 2007; Joseph and Carlson, 2015; Kohl et al., 2015). In both chemosensory systems, the sensory neurons are located in hair-like cuticular structures named sensilla. While little is known about the processes mediating the detection of non-volatile pheromones in gustatory sensilla, studies conducted over the last two decades have considerably elucidated the elements and mechanisms of volatile pheromone signal detection in olfactory sensilla on the antenna (reviewed in Leal, 2013; Kohl et al., 2015; Montagne et al., 2015; Fleischer et al., 2018). The current data indicate a function of pheromone-binding proteins (PBPs) in taking up pheromones from the air and in transferring them across the sensillum lymph toward PRs residing in the ciliary membrane of PRNs. Insects receive olfactory signals through three main families of chemosensory receptor proteins: the odorant receptors (ORs), the gustatory receptors (GRs), and ionotropic receptors (IRs) (Montagne et al., 2015; Wicher, 2015; Fleischer et al., 2018). The large majority of hitherto identified insect PRs are members of the OR family. Additionally, in Drosophila, few GRs and IRs are involved in pheromone reception (Joseph and Carlson, 2015; Kohl et al., 2015). Like OR-expressing olfactory sensory neurons (OSNs) responding to general odorants, proper function of PRNs endowed with a PR type belonging to the OR family is supposed to require the OR co-receptor (Orco). The Orco protein is considered to form heteromeric complexes with ligand-binding ORs and to function as non-selective cation channel (Wicher, 2015; Butterwick et al., 2018; Wicher, 2018). In addition to PBPs and PRs, a CD36-related protein with two transmembrane domains named SNMP1 is necessary for fast and sensitive responses of PRNs (Jin et al., 2008; Li et al., 2014). SNMP1 has been suggested to interplay with PBPs and PRs in pheromone detection (Rogers et al., 2001a; Benton et al., 2007; Nichols and Vogt, 2008), but the mode of interaction is still cryptic.

Based on current data, most notably on Drosophila, complexes of a ligand-binding PR and Orco underlie ionotropic chemo-electrical signal transduction in PRNs (Benton et al., 2006; Ha and Smith, 2009). Yet, recent data obtained from moths indicate that in some insect species, metabotropic processes might be involved and that PRs activate G protein-mediated second messenger cascades, leading to opening of cation channels and depolarization of PRNs (Stengl, 2010; Nolte et al., 2016).

Nearly 70 years after identification of the first insect pheromone in the silkworm moth *Bombyx mori* (Butenandt et al., 1959), enormous progress has been made in understanding the primary processes in the peripheral detection of pheromones. On the molecular level, most notably genes encoding PBPs, PRs, and SNMP1 have been unraveled and deeper insights into the mechanism of the chemo-electrical signal transduction have been obtained. Some fundamental questions have been resolved mostly through studying model insects such as Drosophila and several moth species; however, many issues are a matter of discussion and await further investigation. In this review, we discuss data and concepts regarding the molecular basis of peripheral pheromone reception in insects. We will particularly focus on current knowledge on PRs and the role of olfactory key elements in the peripheral detection, transduction and discrimination of pheromone signals.

## Insect Pheromones – Biological Relevance and Diversity

Per definition, pheromones are chemicals released by an individual and received by conspecifics in which they elicit specific reactions (Karlson and Lüscher, 1959). In insects, pheromones trigger and control various essential behaviors as well as pivotal physiological processes (Yew and Chung, 2015). Insect pheromone communication has fascinated scientists since centuries. The vital importance of a female-released scent for attracting male moths was realized already in the 19th century (Fabre, 1879), but it was not before the late 1950s that the first insect pheromone was chemically unraveled. This was (Z,E)-10,12-hexadecadienol named bombykol, the major component in the sex pheromone emitted by females of the silkworm moth *B. mori* to attract the males (Butenandt et al., 1959). Later bombykal, (Z,E)-10,12-hexadecadienal, was deciphered as second minor constituent of the female sex pheromone (Kaissling et al., 1978). To date, species-specific sex pheromone blends have been described for hundreds of moth species^1^; these pheromones serve as aphrodisiacs and/or attractants to signal the presence of potential mating partners and to indicate their reproductive status and fitness (Yew and Chung, 2015). Full biological activity of the blend is only provided when the components are present in the correct ratio (Vickers et al., 1991; Baker, 2008). Similar to sex pheromones, insects use aggregation pheromones to attract conspecifics; however, both sexes are affected (Wertheim et al., 2005). Aggregation pheromones facilitate cooperative exploitation of rich food sources (Prado and Tjallingii, 1997; Durisko et al., 2014), mate finding (Verhoef and Nagelkerke, 1977), and protection from dangers (Gamberale and Tullberg, 1998; Riipi et al., 2001). Contrary to attracting sex and aggregation pheromones, courtship inhibition pheromones prevent courtship behavior and repel conspecifics (Yew and Chung, 2015). Alarm pheromones, however, can induce dispersal on the one hand but also recruitment of conspecifics and aggression against an opponent on the other hand (Wilson and Regnier, 1971). In eusocial insects (wasps, bees, ants, and termites), pheromones are crucial for the establishment of a social hierarchy as well as suppression of reproduction in workers. Moreover, given pheromones allow kin recognition and may evoke aggression upon detection of foreign pheromone profiles (Yew and Chung, 2015; Leonhardt et al., 2016).

The various functional relevancies of pheromones for insect behavior and physiology are mirrored by the chemical diversity of pheromonal compounds, including hydrocarbons,acetate esters, alcohols, acids, epoxides, ketones, isoprenoids, and triacylglycerides (Yew and Chung, 2015). While some of the pheromonal substances appear to be rather species-specific, others are shared by different insect species (Dewhirst et al., 2010; Roelofs, 2016). Pheromone blends from different species with a partially overlapping composition are typical for sex pheromones released by female moths (de Bruyne and Baker, 2008; Roelofs, 2016) or pheromones allowing kin recognition in eusocial insects (Cervo et al., 2002; Ruther et al., 2002). In these cases, the distinctive combination and ratio of components renders the pheromone species-specific.

With respect to the biological relevance of insect pheromones, it is noteworthy that some of these substances are also detected by respective predatory insects and parasitoids in order to facilitate tracking of their victims. In turn, pheromones released by predatory insects can be received by their insect prey in which they evoke predator avoidance behavior (reviewed by Wyatt, 2014). Moreover, given plants produce and release insect sex pheromone substances to attract insect pollinators (reviewed by Schiestl, 2005). Thus, pheromonal substances can also function as allelochemicals that mediate interspecific communication. Consequently, in terms of chemical ecology, at least some insect pheromone compounds and their detection have a relevance that clearly goes beyond communication with conspecifics.

## Architecture of the Peripheral Pheromone Detection System

**FIGURE 1:**
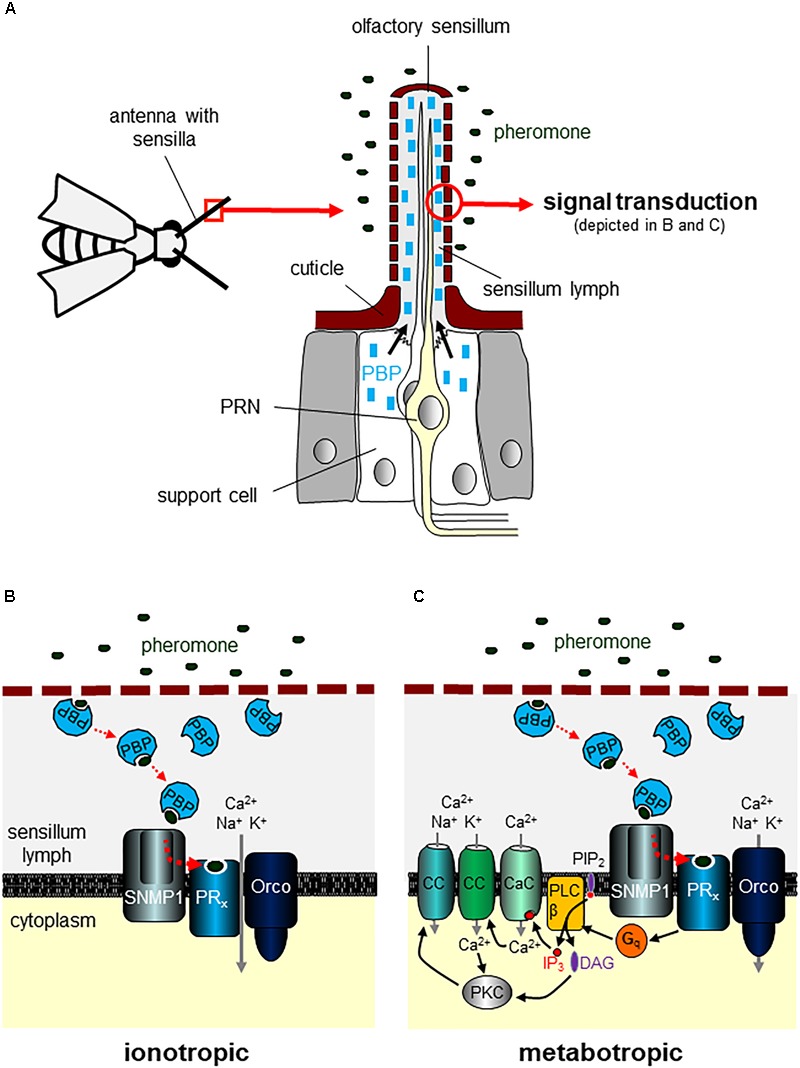
Detection of volatile pheromones on the antenna. **(A)** The antenna of insect carries numerous hair-like extensions of the cuticle termed sensilla. Olfactory neurons extend their ciliary dendrites into the sensillum shaft that is filled with sensillum lymph. A subset of sensilla house pheromone-responsive neurons (PRNs). Support cells associated with the sensory neurons produce pheromone-binding proteins (PBPs) and secrete large quantities of PBPs into the aqueous sensillum lymph. **(B,C)** Volatile pheromones entering the sensillum through cuticle pores are supposed to be taken over by PBPs that solubilize the mostly hydrophobic molecules in the lymph and transfer them to a given pheromone receptor (PR_x_). Different models have been suggested for insect pheromone signal transduction. Based mainly on results from studies using *Drosophila melanogaster* (Sato et al., 2008; Touhara, 2009), a purely ionotropic mechanism has been proposed **(B)**. After reaching the dendritic membrane of a PRN, the ligand-loaded PBP is supposed to interact with the sensory neuron membrane protein 1 (SNMP1). SNMP1 acts as co-receptor mediating the release of pheromones from PBPs and the transfer to the PR_x_ that forms a channel complex with the OR co-receptor (Orco). Binding of the pheromone to the PR_x_ opens the channel complex leading to an influx of cations into the cell. In an alternative model **(C)** mostly based on data from moths, notably the hawk moth *Manduca sexta* (Stengl and Funk, 2013; Nolte et al., 2016), a role of the PR_x_/Orco complex as primary transduction channel is challenged. Instead, pheromone binding to the PR_x_ is supposed to activate a G protein (G_q_)/phospholipase C type β (PLCβ) signaling pathway that via the breakdown of phosphatidylinositol 4,5-bisphosphate (PIP_2_) into inositol 1,4,5-trisphosphate (IP_3_) and diacylglycerol (DAG) induces opening of several ion channels in the plasma membrane. The rise in IP_3_ rapidly opens a calcium-selective ion channel (CaC) evoking increased intracellular Ca^2+^ concentrations. This rapid rise in Ca^2+^ gates Ca^2+^-activated cation channels (CC) and increases the activity of protein kinase C (PKC). PKC is also activated by the rise of DAG. As a result, enhanced PKC activity leads to the opening of further CC.

The detection of pheromones is mediated via chemosensory organs (Wyatt, 2014), although some pheromonal compounds seem to bypass conventional sensory organs and elicit behavioral or physiological responses via directly affecting target tissues (Koene and ter Maat, 2001). The majority of hitherto reported insect pheromones are volatile and detected via OSNs housed in olfactory sensilla (Figure 1A) that are mainly concentrated on the major olfactory organs, the antennae (Hansson and Stensmyr, 2011; Renou, 2014). Yet, in the fruit fly *Drosophila melanogaster*, some pheromones have been reported to stimulate gustatory/taste neurons located in sensilla on the labellum or legs (Lacaille et al., 2007; Miyamoto and Amrein, 2008; Moon et al., 2009; Inoshita et al., 2011). Contrary to aporous and uniporous sensillar types (e.g., mechanosensory and gustatory sensilla), the olfactory sensilla have numerous pores in their cuticle wall, giving pheromones and other odorants from the environment easy access to the inside of a sensillum (Steinbrecht, 1997).

According to their morphology, insect olfactory sensilla are classified into three main categories: coeloconic, basiconic, and trichoid (Steinbrecht, 1996; Stocker, 2001). Electrophysiological recordings from pheromone-responsive trichoid sensilla have revealed that this sensillum type generally comprises 1–3 OSNs (Meng et al., 1989; Almaas and Mustaparta, 1991; Kurtovic et al., 2007); however, in a sensillum with various neurons, not all OSNs necessarily are dedicated to pheromone detection (Almaas and Mustaparta, 1991; Baker et al., 2004). In moths, PRNs are typically clustered in the slender sensilla trichodea (Keil, 1989; Meng et al., 1989). Nonetheless, pheromone detection in insects is not restricted to the trichoid sensillum type. While no PRNs have been located to sensilla coeloconica yet, single sensillum recordings have identified PRNs also among the OSNs of the morphologically different sensilla basiconica. For example, in the desert locust *Schistocerca gregaria*, this sensillum type contains clusters of 20–50 OSNs, some of which respond to the courtship inhibition pheromone phenylacetonitrile (Ochieng’ and Hansson, 1999; Seidelmann and Ferenz, 2002). More recently, in the ants *Ooceraea biroi* and *Harpegnathos saltator*, neurons detecting proposed pheromones were found among a larger number of OSNs located in the female-specific basiconic sensilla on the antennal club (McKenzie et al., 2016; Ghaninia et al., 2017). In contrast, in the beetle *Monochamus galloprovincialis*, basiconic sensilla house only 1–2 OSNs and were reported to contain a PRN tuned to an aggregation pheromone (Alvarez et al., 2015). Overall, the peripheral olfactory system of insects shows a remarkable morphological diversity (Hansson and Stensmyr, 2011) and it remains unclear whether a trichoid or basiconic sensillum architecture confers a functional advantage in detecting a particular class of pheromones.

Some insects show morphological specializations considered as evolutionary adaptation to sensitize pheromone reception. This is particularly obvious in species releasing pheromones in a sex-specific way (for instance moths or beetles). To increase the receptive surface, the antennae of the receiving sex (usually males in moths and females in beetles) are substantially enlarged and equipped with numerous, often strikingly long sensilla dedicated to the detection of pheromonal substances (Meng et al., 1989). A prime example for such a sexual dimorphisms are silk moth species of the genus *Antheraea* where only the males comprise extremely large feather-like antenna endowed with tens of thousands of long trichoid sensilla, most of which receive female-released pheromone components (Schneider et al., 1964; Meng et al., 1989).

## Identification of the First PRs

The chemical diversity and multicomponent composition of pheromones requires highly elaborated sensory systems for the precise detection and discrimination of species-specific pheromones. Typically, insect OSNs are endowed with a single type of olfactory receptor protein (“one receptor per neuron” rule) that confers responsiveness to cognate ligands (Vosshall et al., 2000; Dobritsa et al., 2003; Couto et al., 2005; Hallem and Carlson, 2006). For insect species employing multiple pheromones in chemical communication, this principle implies a larger repertoire of tuned PRNs equipped with distinct PRs.

The initial search for PRs was based on the notion that receptors for volatile pheromones belong to the family of insect ORs. Insect ORs were first identified in *D. melanogaster* (Clyne et al., 1999; Gao and Chess, 1999; Vosshall et al., 1999), providing the basis for the discovery of the first insect PRs in moths 5 years later (Krieger et al., 2004; Sakurai et al., 2004). By applying bioinformatics to screen Drosophila genome sequences for genes encoding proteins structurally related to heptahelical mammalian ORs, a large family of Drosophila OR (DmelOr) genes was found. The corresponding DmelOr proteins were expressed in subsets of OSNs in the antennae and maxillary palps and conferred odorant sensitivity to OSNs (Clyne et al., 1999; Gao and Chess, 1999; Vosshall et al., 1999; Hallem et al., 2004; Hallem and Carlson, 2006). Subsequently, using the DmelOr sequences, bioinformatics and differential screening approaches to search genome and cDNA sequences of moths led to the discovery of OR genes in the tobacco budworm *Heliothis virescens* and the silkmoth *B. mori* (Krieger et al., 2004; Sakurai et al., 2004). In both moth species, a small subfamily of ORs was found to display more than 40% sequence identity, which is strikingly higher than the about 10–20% identity between other insect ORs. In accordance with a role in the detection of female sex pheromone components (SPCs), members of the conserved subfamilies were found to be expressed selectively in OSNs of male pheromone-responsive sensilla trichodea. Furthermore, functional analysis of these ORs in heterologous expression systems confirmed their responsiveness to distinct SPCs (Sakurai et al., 2004; Nakagawa et al., 2005; Grosse-Wilde et al., 2006, 2007; Wang et al., 2011). Matching the predictions from single sensillum recordings of *H. virescens* (Almaas and Mustaparta, 1991; Baker et al., 2004), expression of the OR types HR13 and HR6 responding to the major (HR13) and the minor (HR6) component of the female sex pheromone could be assigned to OSNs of pheromone-responsive trichoid sensilla classified as type A and type B, respectively (Gohl and Krieger, 2006; Grosse-Wilde et al., 2007; Zielonka et al., 2017). Likewise, expression of the *B. mori* receptors BmOR1 and BmOR3 detecting the female-released SPCs bombykol and bombykal, respectively, was localized to the corresponding electrophysiologically characterized pairs of OSNs in sensilla trichodea of male silk moths (Kaissling et al., 1978; Krieger et al., 2005; Nakagawa et al., 2005).

## Reported PRs Across Insects

Since the initial discovery of insect PRs, advances in sequencing technologies and bioinformatics tools have rapidly increased the number of available insect genomes and gave access to the OR gene repertoires of many species. As a consequence, also the list of insects with described PRs has grown continuously. By utilizing homology-based search methods, genes encoding PRs for female SPCs were identified in various lepidopteran species. This was apparently facilitated by the high degree of conservation between moth sex pheromone receptors (SPRs) reflected in the characteristic clustering of moth SPRs in a “SPR clade” in phylogenetic trees of insect ORs (Engsontia et al., 2014; Koenig et al., 2015; Steinwender et al., 2015). However, it is important to recognize that not all ORs that group in the lepidopteran “SPR clade” are necessarily PRs. For example, the receptor types HR14 and HR16 from *H. virescens* mediate responses to pheromone compounds of other species that act as behavioral antagonist in *H. virescens* (Grosse-Wilde et al., 2007; Wang et al., 2011). Similarly, CpomOR3 and CpomOR6 of the codling moth *Cydia pomonella* are activated by a plant-derived odorant and a pheromone antagonist, respectively (Cattaneo et al., 2017).

Beyond lepidopteran SPRs, a number of proven and candidate PRs with proposed roles in various social, sexual, and reproductive behaviors have been reported for dipteran (Ha and Smith, 2006; Kurtovic et al., 2007), hymenopteran (Wanner et al., 2007; Pask et al., 2017), hemipteran (Liu F. et al., 2017; Zhang et al., 2017), and orthopteran species (Pregitzer et al., 2017). Amongst these, AmOR11 of the honey bee *Apis mellifera* (Hymenoptera) was identified as PR for the queen substance 9-oxo-2-decenoic acid (9-ODA) that attracts workers to the queen, inhibits worker ovary development and acts as a sex pheromone by attracting drones during mating flights (Wanner et al., 2007). In the common bedbug *Cimex lectularius* (Hemiptera), several ORs detect different components of the aggregation pheromone (Liu F. et al., 2017). In another hemipteran species, *Acyrthosiphon pisum*, ApisOR5 mediates responses to the aphid alarm pheromone (E)-β-farnesene (Zhang et al., 2017).

Comprehensive studies have been conducted to identify pheromonal compounds and their respective PRs in the powerful genetic model *D. melanogaster* (Diptera) leading to a number of ORs implicated in various pheromone–driven behaviors of the vinegar fly (reviewed in Van der Goes van Naters, 2014; Kohl et al., 2015). DmelOr67d and DmelOr65a were found to detect the male-produced pheromone cis-vaccenyl acetate (cVA) that acts as aphrodisiac in females, inhibits courtship in males and promotes male/male aggression (Kurtovic et al., 2007; Wang and Anderson, 2010; Liu et al., 2011; Pitts et al., 2016). However, recent results indicate that Or65abc-expressing neurons are unresponsive to cVA (Pitts et al., 2016), challenging a role of DmelOr65a in cVA detection. DmelOr7 has been described to detect (Z)-9-tricosene, a pheromone released by males guiding aggregation and oviposition decisions in females (Lin et al., 2015). Receptors DmelOr69aB and DmelOr69aA are tuned to the pheromone (Z)-4-undecenal (Z4-11Al) that is released by female flies and induces flight attraction in both sexes. Intriguingly, these PR types are also activated by food odorants (Lebreton et al., 2017). In addition, DmelOr88a and DmelOr47b have been reported as PRs for fly–produced fatty acid methyl esters mediating copulation and attraction (Dweck et al., 2015); however, a conflicting study found little direct impact of the respective OSNs on courtship behaviors; instead, responses of these OSNs to a number of non-fly odors were observed (Pitts et al., 2016).

In Drosophila, also other receptors than ORs are considered as PRs. Notably, a small number of heptahelical GRs as well as members of the so-called pickpocket (Ppk) subfamily of degenerin-epithelial sodium channels (Deg-ENaCs) are required for pheromone-guided sexual behaviors (reviewed in Joseph and Carlson, 2015; Kohl et al., 2015). Neurons expressing these GRs and Deg-ENaCs are activated by cuticular hydrocarbons (CHCs) produced by either one or both sexes. Some of the identified CHCs have been shown to be volatile (Farine et al., 2012) suggesting sensory detection of pheromonal CHCs through the olfactory system as well as the taste system. While the Drosophila olfactory system seems to have some relevance for detecting volatile CHCs (Farine et al., 2012), all GRs and Deg-ENaCs implicated in the detection of pheromonal CHCs are expressed in neurons of gustatory sensilla on the labellum and the legs/tarsi (Bray and Amrein, 2003; Miyamoto and Amrein, 2008; Moon et al., 2009; Jeong et al., 2013). Thus, in Drosophila, pheromonal CHCs appear to be primarily sensed through contact chemoreception and the taste system. Interestingly, and contrary to the vinegar fly, in the ant *H. saltator*, a subfamily of ORs that is highly expressed in the antenna detects different CHCs supposed to be important in mediating eusocial behavior, including a candidate queen pheromone component (Pask et al., 2017; Slone et al., 2017). This finding suggests an outstanding importance of the ant olfactory system for the detection of CHC pheromones and may indicate different evolutionary adaptation to the detection of pheromonal CHCs in insects.

## Special Aspects of PR Expression

In insect species in which only one sex releases SPCs, PR expression is often biased, with exclusive or predominant expression in the non-releasing sex. This is particularly evident for PRs detecting the major component of the female-released sex pheromone blend in moths; these PRs are mainly expressed by males. In contrast, but in accordance with cVA-controlled behavior in both sexes of *D. melanogaster*, no sexual dimorphism in the expression was found for the receptor DmelOr67d detecting the male-released pheromone cVA (Kurtovic et al., 2007). Interestingly, cVA evokes in a DmelOr67d-dependent manner opposite behaviors in males versus females: while cVA elicits suppression of courtship in male flies, it promotes mating behavior in females (Ejima et al., 2007; Kurtovic et al., 2007; Datta et al., 2008).

Similar to Drosophila males, behavioral and electro-physiological studies provide evidence for “autodetection” of pheromones by females of various moth species, i.e., detection of SPCs released by themselves (Holdcraft et al., 2016). Contrary to what the term “autodetection” suggests, this ability presumably does not mainly serve detection of the compounds released by the pheromone-producing individual itself but rather the detection of SPCs released by conspecific females in the surrounding. Thus, sex pheromone information may be used by females to avoid places of high mating competition and unfavorable oviposition sites, thereby minimizing competition for ecological resources (Harari and Steinitz, 2013; Holdcraft et al., 2016). In line with this notion, expression of PRs for female-released SPCs has been reported for the antennae of female moths (Bengtsson et al., 2012; Sun et al., 2013; Holdcraft et al., 2016; Zielonka et al., 2017). For example, in female antennae of *H. virescens* that comprise trichoid sensilla tuned to the female-released SPC (Z)-9-tetradecenal (Hillier et al., 2006), OSNs expressing the cognate PR type HR6 are located to this sensillum type (Zielonka et al., 2017).

Intriguingly, in recent studies of several moth species, antennal OSNs of larvae were found to respond to female SPCs; moreover, the caterpillars were also attracted to food sources that contain such SPCs, suggesting that sex pheromones might serve as a relevant cue for larvae in food source selection (Poivet et al., 2012; Jin et al., 2015; Zhu et al., 2016). While in *Spodoptera littoralis*, PBPs but no respective SPRs were identified in the larval antenna (Poivet et al., 2012, 2013), analysis of the sensilla from *H. virescens* caterpillars revealed responses to SPCs and expression of the PR types HR6 and HR13 for the major and the minor SPC in distinct OSNs of basiconic sensilla. In addition, co-expression of PRs with SNMP1 and expression of PBPs was found (Zielonka et al., 2016). This finding indicates that in moths, the responsiveness to pheromones in larval sensilla is based on the same molecular machinery as in the antenna of adults. However, the biological relevance of pheromone detection in the antenna of larvae needs further investigation.

Noteworthy, in moths, expression of SPRs is not confined to the antenna since RNA encoding these receptors has also been found in abdominal tissue from both sexes (Krieger et al., 2004; Widmayer et al., 2009). Detailed analyses of the abdomen from females of *H. virescens* have shown that HR6 and HR13 are expressed in sensilla surrounding the tip of the ovipositor. These findings have led to speculations that SPRs in the female abdomen might be involved in feedback mechanisms controlling the release of SPCs from pheromone glands (Widmayer et al., 2009).

## Ligand Specificity of PRs

Ongoing collaborative projects like the I5 K initiative that intends to sequence the genomes of 5000 arthropods will give access to the OR, IR, and GR gene repertoires and thus to candidate PR sequences of hundreds of nominated insect species. However, identification of PRs among the plethora of predicted olfactory receptor proteins in a given species will be a big challenge and will not only require detailed knowledge of pheromones but also appropriate and powerful functional expression systems for receptor deorphanization. Hitherto, three main *in vivo* heterologous expression systems are available, all of which have been applied successfully for PR characterization. These are (i) Xenopus oocytes coupled to voltage–clamp electrophysiology, (ii) mammalian or insect cell lines coupled to calcium imaging, and (iii) the so-called Drosophila “empty neuron” and T1 sensillum systems in combination with electrophysiological single sensillum recordings (Nakagawa et al., 2005; Grosse-Wilde et al., 2006; Kurtovic et al., 2007; Mitsuno et al., 2008; Forstner et al., 2009; Syed et al., 2010; Wanner et al., 2010; Zhang et al., 2016; Pask et al., 2017; reviewed in Montagne et al., 2015; Fleischer et al., 2018). In addition, a cell-free functional expression system involving OR synthesis in giant vesicles and patch clamp recordings has been reported (Hamada et al., 2014).

For the assessment of candidate PRs (and other ORs), the Xenopus oocyte system has been most widely applied (Nakagawa et al., 2005; Sun et al., 2013; Zhang et al., 2015, 2016; Liu F. et al., 2017). SPRs from moths functionally expressed in frog oocytes displayed a wide range of ligand specificities with receptors tuned to a single or to several components of female sex pheromones (Mitsuno et al., 2008; Wanner et al., 2010; Wang et al., 2011). Congruent results were described for moth SPRs analyzed in human embryonic kidney (HEK) cells (Grosse-Wilde et al., 2006, 2007; Forstner et al., 2009). Similarly, characterization of ORs of the common bedbug *C. lectularius* in frog oocytes revealed several ORs with partly overlapping tuning properties for distinct compounds of the multicomponent aggregation pheromone (Liu F. et al., 2017).

Candidate PRs of the ant *H. saltator* were characterized using the Drosophila “empty neuron” system (Pask et al., 2017). Systematic testing with a diverse panel of hydrocarbons revealed that most receptors are narrowly tuned, suggesting that in ants several PRs contribute to the detection and discrimination of different CHCs.

The Drosophila T1 sensillum system makes use of a given OSN type endogenously expressing the PR DmelOR67d (Ha and Smith, 2006), thus providing a sensillum environment and an OSN type equipped for pheromone detection. On the molecular level, this includes expression of a pheromone-transporting PBP and of SNMP1 shown to be required for proper function of Drosophila and moth PRs (Benton et al., 2007; Jin et al., 2008; Pregitzer et al., 2014). Several studies have proven the suitability of the Drosophila T1 sensillum for the characterization of PRs of other insects. For instance, replacement of DmelOr67d by the OR types SlitOR6 of *S. littoralis* (Montagne et al., 2012), BmOR1 of *B. mori* (Syed et al., 2010), or HR13 of *H. virescens* (Kurtovic et al., 2007) allowed to validate these ORs as narrowly tuned PRs for distinct SPCs.

Functional analyses using heterologous expression systems are the preferred tools for the assessment of the ligand specificity of insect PRs. Yet, whether the concentration of the stimulus experimentally applied in functional analyses of PRs complies with the natural pheromone concentrations detected by an insect is mostly unclear; however, it is a critical parameter for assessing the tuning of PRs. In addition, other experimental factors such as the set of compounds tested may affect the assessment of the receptor tuning (reviewed in Andersson et al., 2015). For PRs concluded to mediate the detection of several pheromonal compounds, a further aspect should be considered. In heterologous systems, the assessment of ligand specificities of PRs is usually conducted in the absence of the endogenous PBPs. Importantly, in the cases where PBPs have been employed in functional analyses of PRs in the Xenopus oocyte or the HEK cell system, more sensitive and specific responses were obtained (Grosse-Wilde et al., 2007; Forstner et al., 2009; Sun et al., 2013). This finding suggests that “ligand-matched” pairs of PBPs and PRs appear to underlie the overall reactivity of a pheromone detection system (Grosse-Wilde et al., 2006, 2007; Forstner et al., 2009). Moreover, PBPs appear to be far more than just solubilizers and transporters but also function as pre-filters enabling only distinct compounds to reach a PR. Thus, for a given PR classified as “broadly tuned” based on functional analyses in the absence of endogenous PBPs, the determined ligand spectrum may contain compounds that the PR protein in the ciliary membrane of a respective PRN would never face under natural conditions.

The existing data indicate that insects employ a range of narrowly and broadly tuned PRs for the detection of multicomponent pheromone blends and suggest combinatorial coding as the primary coding principle to perceive complex pheromone signals. Consequently, the pheromone detection process is not fundamentally different from the mechanisms insects use to analyze complex mixtures of odors originating from food sources, hosts or oviposition sites; these processes also employ specifically and broadly tuned ORs to detect and discriminate relevant odorants (Hallem and Carlson, 2006; Carey et al., 2010; Wang et al., 2010; Dweck et al., 2013).

In the current concept of pheromone detection, PRs that are specifically responsive to a single pheromone compound confer the ability to distinguish chemically very similar compounds, such as SPCs with different fatty acid chain length, same molecular backbone but different functional groups or stereoisomeric compounds (Zhang and Löfstedt, 2015). In male moths, narrowly tuned SPRs are crucial to discriminate conspecific sex pheromones from related molecules co-existing in the environment; thus, they are essential for precise mate recognition. The role of moth SPRs with a broader response spectrum and the determinants of their ligand selectivity are largely unclear. Probably, they represent a preadaptation to ensure effective tracking of female-released sex pheromone signals even if the composition of the blend undergoes slight changes (Gould et al., 2010; Heckel, 2010; Zhang and Löfstedt, 2015). Noteworthy, recent analyses of the SPR orthologs HassOR14b and HarmOr14b from the closely related moth species *Helicoverpa assulta* and *Helicoverpa armigera* showed that only few key amino acid residues appear to be sufficient to shift the ligand specificity between orthologous but differently tuned SPRs. In contrast, substitution of many other amino acid residues had no or only subtle effects (Yang et al., 2017). Based on these findings, it has been suggested that consecutive point mutations in key amino acids of SPRs during evolution may have been major drivers in the course of speciation.

## Transduction Mechanisms and Interplay of PRs with Other Signaling Proteins

### PBPs and Their Relevance for Pheromone Transport and Detection

Pheromone detection is initiated when pheromonal substances enter olfactory sensilla via cuticular pores. In the aqueous lymph, pheromonal and odorous molecules bind to water-soluble odorant-binding proteins (OBPs) synthetized and released by support cells that surround OSNs (Figure 1A). OBPs are supposed to mediate solubilizing and subsequent transport to the relevant olfactory receptor proteins residing in the dendritic membrane of OSNs. For the specific binding and transport of pheromones to PRs, a subfamily of the OBPs, the PBPs, is regarded as essential. During the last decades, numerous PBPs from various insect species have been identified. For a more detailed review of PBPs, the reader is referred to articles that explicitly highlight this group of proteins (Pelosi et al., 2006; Fan et al., 2011; Vieira and Rozas, 2011; Leal, 2013; Brito et al., 2016). In brief, *in vitro* and *in vivo* studies using PRs from different moth species and the cognate pheromone compounds have demonstrated that the sensitivity as well as the specificity of pheromone-evoked signaling is enhanced in the presence of appropriate PBPs (Grosse-Wilde et al., 2006; Forstner et al., 2009; Chang et al., 2015). These observations are consistent with a role of PBPs in solubilizing and transporting pheromones. Yet, the precise role of PBPs for pheromone detection is uncertain. In this regard, it has been reported that in Drosophila flies mutant for the PBP type LUSH (OBP76a), the responsiveness to the LUSH-binding pheromone cVA is abolished and the spontaneous activity in cVA-sensitive antennal OSNs in the absence of the pheromone is diminished (Xu et al., 2005). Introducing recombinant LUSH protein directly into cVA-responsive sensilla from LUSH mutant flies restored spontaneous activity (Xu et al., 2005). Moreover, detailed analyses indicate that LUSH is an inactive, extracellular ligand that is converted by cVA into an activator of PRNs (Laughlin et al., 2008). However, in a more recent study, activation of the relevant PRNs was induced by higher concentrations of cVA even in the absence of LUSH (Gomez-Diaz et al., 2013). Consequently, further studies are required to unravel the functional role of LUSH (and other PBPs) for pheromone detection.

### Role of SNMP1 in Pheromone Detection

Besides an interplay of PRs and PBPs, a number of studies have demonstrated that in insects, sensitive pheromone signaling requires SNMP1 (Benton et al., 2007; Jin et al., 2008; Li et al., 2014; Pregitzer et al., 2014). SNMP1 was first discovered in the moth *Antheraea polyphemus* as a prominent protein in the dendritic membrane of PRNs (Rogers et al., 1997, 2001b). More recent studies revealed co-expression of several proven and candidate PRs with SNMP1 in OSNs (Krieger et al., 2002; Benton, 2007; Pregitzer et al., 2014, 2017) and suggest a localization of SNMP1 in close proximity to receptor proteins in the membrane (Benton et al., 2007; German et al., 2013). In cells expressing PRs, SNMP1 is required for highly sensitive responses and is important for rapid activation as well as termination of pheromone-induced activity (Benton, 2007; Li et al., 2014; Pregitzer et al., 2014). The specific function of SNMP1 in the pheromone signaling process is unclear. Already early, a role as co-receptor that may be involved in unloading pheromones from PBPs and passing the signal molecules to PRs has been postulated (Rogers et al., 1997; Vogt et al., 2009). This concept has recently been substantiated by demonstrating that SNMP1 may indeed bind pheromones to its large ectodomain and may forward ligands through this tunnel-like domain to a PR (Gomez-Diaz et al., 2016). How SNMP1 interplays with PRs and PBPs is an open question. Furthermore, it is unknown whether and to what extend SNMP1 might be also involved in the sensitive detection of non-pheromonal compounds.

### Ionotropic Versus Metabotropic Transduction Processes in Pheromone-Induced Signaling and the Uncertain Role of the OR Co-receptor Orco

Insect OSNs positive for ORs – including PRs – seem to commonly co-express a non-canonical member of the OR family designated as Or83b or Orco (Krieger et al., 2003; Larsson et al., 2004; Pitts et al., 2004; Jones et al., 2005). Over the past years, substantial evidence has been accumulated that Orco forms multimers of unknown stoichiometry with ORs and that Orco is crucial for dendritic localization, membrane targeting, and subsequent signaling of ORs (Larsson et al., 2004; Neuhaus et al., 2005; Benton et al., 2006). While no structural model for OR/Orco heteromers exists yet, the structure of Orco homomers has been elucidated recently by cryo-electron microscopy. The structure indicates a channel architecture, with four subunits symmetrically arranged around a central pore (Butterwick et al., 2018). With respect to pheromone detection, deletion or silencing of Orco expression has been reported to evoke a dramatic loss of OSN responsiveness to pheromonal compounds (Koutroumpa et al., 2016; Li et al., 2016; Liu Q. et al., 2017). Consistently, activation of PRs by appropriate pheromones in heterologous expression systems is also significantly higher upon co-expression of Orco (Nakagawa et al., 2005; Wanner et al., 2007). In fact, co-expression of the *B. mori* bombykol receptor BmOR1 with Orco in heterologous expression systems induced a considerable ligand-stimulated non-selective cation channel activity (Nakagawa et al., 2005; Sato et al., 2008). In this context, in addition to its function as a chaperon, in experiments using heterologous expression, Orco has been identified as a spontaneously opening Ca^2+^-permeable and unspecific cation channel (Wicher et al., 2008; Jones et al., 2011; Nolte et al., 2013). Thus, such observations have led to the concept that heteromeric complexes comprising Orco and ORs/PRs function as ligand-gated ion channels in which the binding of the ligand is exclusively mediated by the OR/PR protein (Figure 1B; Sato et al., 2008; Touhara and Vosshall, 2009; Wicher, 2015). Yet, the functional relevance of Orco in PRNs is still a matter of controversial discussion. In spite of the above described findings related to PRs from bees, *D. melanogaster* and *B. mori*, recent studies with PRs and/or PRNs from different moth species (including *H. virescens* and *Manduca sexta*) challenge the notion that pheromone-evoked signaling in OSNs is ionotropic and relies on Orco. Notably, in HEK cells expressing PRs but lacking Orco, pheromones elicited clear responses (Grosse-Wilde et al., 2006, 2007; Forstner et al., 2009). Furthermore, experimental findings of tip recordings from *M. sexta* pheromone-sensitive sensilla upon application of Orco agonists and antagonists argue against an involvement of Orco and ionotropic signaling in the primary transduction processes of pheromone detection. Instead, Orco seems to serve as a slower, second messenger-gated pacemaker channel that controls the membrane potential and hence affects the threshold and kinetics of pheromone-induced responses via changes of intracellular Ca^2+^ baseline concentrations (Nolte et al., 2013, 2016). Although the transduction cascade underlying pheromone-evoked signaling in moth OSNs is still elusive, it has been suggested that this process is largely metabotropic (Stengl and Funk, 2013; Nolte et al., 2016). This notion is in line with the observation that insect ORs – alike G protein-coupled receptors (GPCRs) – are heptahelical receptors although they share no sequence similarities with canonical GPCRs and show an inverted membrane topology with an intracellular N-terminus (Benton et al., 2006; Smart et al., 2008; Tsitoura et al., 2010). Consistent with the heptahelical structure of ORs/PRs and potential downstream G protein-mediated signaling, earlier findings have revealed the synthesis of the second messenger substance inositol 1,4,5-trisphosphate (IP_3_) in insect antennal tissue following exposure to pheromones (Boekhoff et al., 1990). Intriguingly, perfusing *M. sexta* OSNs with IP_3_ elicits a specific sequence of currents that is mimicked by exposure to pheromones (Stengl et al., 1992; Stengl, 1993; Stengl and Funk, 2013). Based on these findings, it has been proposed that in moth OSNs, pheromones elicit via PRs, G proteins, and phospholipase C type β (PLCβ) an increased formation of the second messengers IP_3_ and diacylglycerol, leading to the activation of IP_3_-gated Ca^2+^ channels and Ca^2+^-activated cation channels (Stengl and Funk, 2013). Additionally, activation of protein kinase C (PKC) by diacylglycerol might induce opening of PKC-activated cation channels (Figure 1C). Yet, future studies are urgently required to elucidate in more detail the metabotropic and ionotropic processes that underlie pheromone-evoked signaling in insect OSNs.

The electrical activity evoked in antennal OSNs upon binding of pheromones (or general odorants) to cognate olfactory receptors is transformed into a pattern of action potentials and transmitted via their axons to the primary olfactory center in the brain, a region of the insect deutocerebrum known as the antennal lobe (Martin et al., 2011; Renou, 2014). The axonal terminals of OSNs expressing a distinct PR (or other OR) converge on a single out of numerous spherical units called glomeruli, suggesting a receptor-based map of olfactory connectivity and coding (Vosshall et al., 2000; Couto et al., 2005; Sakurai et al., 2014). The size of the glomeruli appears to correlate with the number of OSNs expressing given receptors in the antennae (Grabe et al., 2016). This is most obvious in male moths comprising particular high numbers of antennal OSNs expressing PRs for female-released SPCs. Accordingly, in the antennal lobe, sex-specific clusters of enlarged glomeruli are found (termed macroglomeruli) that form the so-called macroglomerular complex (Hansson et al., 1992; Christensen and Hildebrand, 2002; Berg et al., 2014). Enlarged and male-specific glomeruli are not restricted to moths. Macroglomeruli have been also reported for bees, ants, and cockroaches (reviewed in Hansson and Anton, 2000; Galizia and Rossler, 2010); consequently, axonal convergence of OSNs endowed with PRs for sex pheromones on macroglomeruli might be a widespread trait in insects.

Yet, although the axonal projection of an individual OSN to a given glomerulus is apparently associated with the olfactory receptor expressed by this neuron, “normal” expression of an OR/PR type is not required to navigate the axon to its target glomerulus (Dobritsa et al., 2003; Wang et al., 2003; Sakurai et al., 2015). This finding indicates that it is not the receptor protein that determines targeting to the appropriate glomerulus; an observation that is in marked contrast to the vertebrate olfactory system (Komiyama and Luo, 2006).

## Conclusion and Future Directions

Research conducted in recent years has greatly advanced our understanding of the cellular and molecular processes that underlie pheromone reception in insects, but at the same time raised many open questions that will stimulate future investigations. On the molecular level, in a number of insects, an array of narrowly and broadly tuned PRs have been identified that mediate the recognition and coding of pheromone signals. In the coming years, genome sequencing and bioinformatics will give access to the olfactory receptor repertoires of a plethora of insect species that use pheromone communication. This will open the avenue to the discovery of PRs and other elements of pheromone reception in species that have yet not been accessible for a molecular analysis. In addition to PRs, current data indicate a crucial role of SNMP1 and PBPs in pheromone reception; however, how these proteins interplay in the process of pheromone signal recognition remains to be determined. To the same extent, the elements and mechanisms of pheromone signal transduction await further illumination, in particular with regard to the debated question whether distinct insect species use ionotropic, metabotropic, or both signaling processes to transduce intraspecific signals.

## Author Contributions

All authors listed have made a substantial, direct, and intellectual contribution to the work, and approved it for publication.

## Conflict of Interest Statement

The authors declare that the research was conducted in the absence of any commercial or financial relationships that could be construed as a potential conflict of interest.

## Footnotes

1 www.pherobase.com

## Abbreviations

CHC(s): cuticular hydrocarbon(s)
cVA: *cis*-vaccenyl acetate
Deg-ENaCs: degenerin-epithelial sodium channels
DmelOr: odorant receptor from *Drosophila melanogaster*
GR(s): gustatory receptor(s)
HEK: human embryonic kidney
IP_3_: inositol 1,4,5-trisphosphate
IR(s): ionotropic receptor(s)
OBPs: odorant-binding proteins
OR(s): odorant receptor(s)
Orco: OR co-receptor
OSN(s): olfactory sensory neuron(s)
PBP(s): pheromone-binding protein(s)
PKC: protein kinase C
PLCβ: phospholipase C type β
PR(s): pheromone receptor(s)
PRN(s): pheromone-responsive neuron(s)
SNMP1: sensory neuron membrane protein 1
SPC(s): sex pheromone component(s)
SPR(s): sex pheromone receptor(s)
